# Hospital-acquired pneumonia is associated with deficient γc-cytokine gene expression

**DOI:** 10.1186/cc9603

**Published:** 2011-03-11

**Authors:** M White, R McManus, T Ryan

**Affiliations:** 1 St James Hospital, Dublin, Ireland; 2 Trinity College, Dublin, Ireland

## Introduction

Lymphocyte homeostasis is dependent on the γc cytokines. We hypothesised that infection in humans is associated with differential gene expression of the γc cytokines and their associated apoptosis mediators.

## Methods

Sixty patients undergoing elective lung resection surgery were recruited. Nineteen patients developed postoperative pneumonia. Pneumonia was diagnosed by CDC NNIC criteria. Gene expression in peripheral blood leukocytes (PBLs) of IL-2, IL-7, IL-15 and IFNγ, Bax, Bim, Bcl-2 was determined by qRT-PCR preoperatively and again on day 1 and day 5 postoperatively. IL-2 and IL-7 serum protein levels were determined by ELISA preoperatively and again on day 1 and day 5 postoperatively.

## Results

In lung resection surgery patients, postoperative pneumonia was associated with a perioperative decrease in IL-2 mRNA (*P *< 0.0001) and IL-7 mRNA (*P *= 0.003). IL-15 gene expression was similar between both groups at all three points. Bcl-2 and Bax gene expressions were similar between both pneumonia and nonpneumonia groups at all three time points. Bim gene expression was greater in the pneumonia group compared with the nonpneumonia group on day 5 postoperatively (*P *= 0.04). IL-2 protein levels were similar in pneumonia and nonpneumonia groups. IL-7 protein levels were similar in all groups.

## Conclusions

Patients with postoperative pneumonia display deficient IL-2 and IL-7 gene expression in PBLs. Aberrant cytokine gene expression may precede the onset of infection.

